# S75. KEYNOTE LECTURE: Basic concept for the understanding of cancer mediated immune rejection

**DOI:** 10.1186/2051-1426-2-S2-I13

**Published:** 2014-03-12

**Authors:** F Marincola, E Wang

**Affiliations:** 1 Sidra Medical and Research Center, Doha, Qatar; 2 Sidra Medical and Research Center, Research Branch, Doha, Qatar

## Background

Cancer is a multi-genic, complex biological phenomenon and its growth is affected by various categories of factors including the genetics of the host, the accumulating genetic alteration within cancer cells and environmental modifiers.

## Material and methods

Therefore, to identify the determinants of immune-mediated tumor rejection during immunotherapy, a systematic and comprehensive monitoring approach needs to be applied that covers the various categories at a time relevant to the therapeutic intervention. In addition, a temporal dimension needs to be added to evaluate in real time the changes induced by treatment and their relationship with clinical outcome.

## Results

In this presentation, we will review the salient concepts that should guide the future monitoring of clinical trials taking advantage of novel and comprehensive technological advances presenting examples of integrated approaches for the assessment of patients' response to adoptive T cell therapy.

## Conclusions

Our experience highlights the need to apply multi-parametric approaches for the understanding of the mechanism(s) leading to cancer rejection by the immune system in humans.

